# A Lactococcal Phage Protein Promotes Viral Propagation and Alters the Host Proteomic Response During Infection

**DOI:** 10.3390/v12080797

**Published:** 2020-07-24

**Authors:** Marie-Laurence Lemay, Sandra Maaß, Andreas Otto, Jérémie Hamel, Pier-Luc Plante, Geneviève M. Rousseau, Denise M. Tremblay, Rong Shi, Jacques Corbeil, Stéphane M. Gagné, Dörte Becher, Sylvain Moineau

**Affiliations:** 1 Département de Biochimie, de Microbiologie, et de Bio-Informatique, Faculté des Sciences et de Génie and PROTEO, Québec City, QC G1V 0A6, Canada; marie-laurence.lemay.1@ulaval.ca (M.-L.L.); jeremie.hamel.1@ulaval.ca (J.H.); Genevieve.Rousseau@greb.ulaval.ca (G.M.R.); rong.shi@bcm.ulaval.ca (R.S.); Stephane.Gagne@bcm.ulaval.ca (S.M.G.); 2 Groupe de Recherche en Écologie Buccale, Faculté de Médecine Dentaire, Université Laval, Québec City, QC G1V 0A6, Canada; Denise.Tremblay@greb.ulaval.ca; 3 Institute of Microbiology, University of Greifswald, 17489 Greifswald, Germany; sandra.maass@uni-greifswald.de (S.M.); andreas.otto@agilent.com (A.O.); dbecher@uni-greifswald.de (D.B.); 4 Institut de Biologie Intégrative et des Systèmes, Université Laval, Québec City, QC G1V 0A6, Canada; 5 Centre de Recherche en Infectiologie de L’Université Laval, Axe Maladies Infectieuses et Immunitaires, Centre de Recherche du CHU de Québec-Université Laval, Québec City, QC G1V 4G2, Canada; pier-luc.plante.1@ulaval.ca (P.-L.P.); jacques.corbeil@fmed.ulaval.ca (J.C.); 6 Centre de Recherche en Données Massives, Université Laval, Québec City, QC G1V 0A6, Canada; 7 Félix D’Hérelle Reference Center for Bacterial Viruses, Université Laval, Québec City, QC G1V 0A6, Canada; 8 Département de Médecine Moléculaire, Faculté de Médecine, Université Laval, Québec City, QC G1V 0A6, Canada

**Keywords:** phages, *Skunavirus*, *Lactococcus lactis*, non-structural phage proteins, phage-host interactions, bacterial proteomes, phage-resistant mutants

## Abstract

The lactococcal virulent phage p2 is a model for studying the *Skunavirus* genus, the most prevalent group of phages causing milk fermentation failures in cheese factories worldwide. This siphophage infects *Lactococcus lactis* MG1363, a model strain used to study Gram-positive lactic acid bacteria. The structural proteins of phage p2 have been thoroughly described, while most of its non-structural proteins remain uncharacterized. Here, we developed an integrative approach, making use of structural biology, genomics, physiology, and proteomics to provide insights into the function of ORF47, the most conserved non-structural protein of unknown function among the *Skunavirus* genus. This small phage protein, which is composed of three α-helices, was found to have a major impact on the bacterial proteome during phage infection and to significantly reduce the emergence of bacteriophage-insensitive mutants.

## 1. Introduction

Phages are abundant in all ecosystems [1,2] and are thought to play a pivotal role in controlling bacterial populations. Their unequalled diversity partly explains why so many of their proteins have no known function [3,4]. An added challenge to studying phage proteins is the network-like complexity of phage–bacteria interactions. Phage structural proteins are found within the virion structure and the current information on these proteins far exceeds that of non-structural viral proteins because they are relatively more conserved, abundant and are easier to study directly on the virion particles using techniques such as SDS-PAGE, mass spectrometry, and electron microscopy [5]. Conversely, non-structural phage proteins are produced, sometimes at low levels, inside the bacterial host where they presumably also interact with various components of the cell machinery. To gain insight into these proteins, a multidisciplinary approach is likely required.

**Figure 1:**
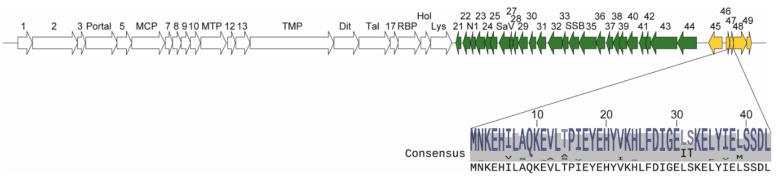
Phage p2 genome, *orf47* location and ORF47 consensus sequence. The genes are represented by arrows and numbers. Early-expressed genes are in green, middle-expressed in yellow and late-expressed in white. The consensus amino acid sequence of ORF47 in 184 publicly available *Skunavirus* genomes is shown (see Figure S1 (Supplementary Materials) for alignment). MCP: Major capsid protein, MTP: Major tail protein, TMP: Tail tape measure protein, Dit: Distal tail protein, Tal: Tail associated lysin, RBP: Receptor binding protein, Hol: Holin, Lys: Lysin, SaV: Sensitivity to AbiV protein, SSB: Single stranded binding protein.

Lactococcal phages belonging to the *Skunavirus* genus are the most endemic and problematic bacterial viruses in the cheese industry worldwide [6]. They are strictly lytic (virulent) and are part of the Siphoviridae family of the order Caudovirales [7]. Because they are often found in raw milk and are hardly affected by pasteurisation, these lytic phages pose a significant risk to the cheesemaking process by infecting and killing industrial strains of *Lactococcus lactis*, which are added to milk to control the fermentation process. Phage p2 is a member of the *Skunavirus* genus and is one of the few models used to study the biology of phages infecting Gram-positive bacteria. It infects the well-studied laboratory strain *L. lactis* MG1363, a plasmid-free derivative of the dairy starter strain NCDO712 [8,9]. Phage p2 dsDNA genome consists of 27,595 bp and 50 annotated open reading frames (ORFs) [10]. The ORFs are organized into three large gene clusters, chronologically transcribed upon infection (Figure 1).

Early- and middle-expressed genes mainly encode for non-structural proteins that are responsible for hijacking the cellular machinery, while most late-expressed genes encode phage structural proteins. Phage p2 structural proteins have been analyzed in great detail, leading to the determination of its complete virion structure using single-particle electron microscopy [11]. However, most of its non-structural proteins are still uncharacterized.

ORF47 of phage p2 is a middle-expressed non-structural protein of 43 amino acids. It is the most conserved non-structural protein within the *Skunavirus* genus [12], but has no known function. In a previous study, we used CRISPR-Cas9 to knock out gene *orf47* and confirmed gene disruption in a functional mutated phage (p2∆47) through whole genome sequencing, confirming its non-essentiality for the infection of *L. lactis* MG1363 under laboratory conditions [13].

To characterize the structural properties of ORF47, the protein was first synthesized and then studied through nuclear magnetic resonance (NMR). We also analyzed the impact of *orf47* deletion (∆47) on phage biology by comparing the lytic cycle of the mutant phage p2∆47 with the wild-type phage p2. We challenged *L. lactis* MG1363 with phage p2∆47 or p2 with the aim of comparing the frequencies of bacteriophage-insensitive mutants (BIMs) as the ability of a bacterial population to adapt and resist phage infection is a key natural phenomenon influencing phage–host dynamics. We investigated the type of resistance mechanisms in BIMs obtained after being challenged with phage p2∆47 or p2. Furthermore, we used label-free quantitative proteomics to compare the proteotypes of *L. lactis* MG1363 infected by either phage p2∆47 or p2 [10]. We showed that the presence of ORF47 prevents *L. lactis* MG1363 to rapidly adapt and become resistant to phage p2. In the absence of ORF47, the phage induces a dormancy-like state in the infected bacterial host.

## 2. Materials and Methods

### 2.1. NMR Structure Determination

The phage protein ORF47 was synthesized by Peptide 2.0 (95.97% purity) and was received lyophilized. NMR sample conditions were: 0.5 mM protein, 20 mM PO_4_, 50 mM KCl, 0.02% azide, pH 6.3, 30 °C, 90% H_2_O:10% D_2_O. All NMR data were recorded on a Varian Inova 600 MHz NMR spectrometer equipped with a triple-resonance XYZ-gradient probe. The following two-dimensional NMR spectra were recorded: 2D wgCOSY, 2D wgNOESY (mixing time of 200 ms), 2D wgTOCSY (mixing times of 50 and 100 ms), natural abundance 2D ^15^N-HSQC, and natural abundance 2D ^13^C-HSQC. NMR spectra were processed with NMRPipe (Supplementary Figures S2 and S3) [14]. Resonance assignments were completed using NMRViewJ (One Moon Scientific, Inc.). Completion of resonance assignments were as follows: 92% for ^1^H, 87% for ^13^Cα/^13^Cβ, and 72% for ^15^N. Thirty-three pairs of phi/psi dihedral angle restraints were derived from TALOS-N [15] using ^1^Hα, ^13^Cα and ^13^Cβ chemical shifts. Final NOE data included 1274 peaks (intraresidue: 485, sequential: 185, short-range: 670, long-range: 63). One hundred structures were calculated using CYANA 3.97, and the best 20 structures were selected. The final 20 structures had no dihedral restraint violation, and one distance restraint violation (mean 0.09 Å, maximum 0.23 Å). The average RMSD for residues 3–39 were 0.43 ± 0.10 Å (backbone) and 0.87 ± 0.12 Å (heavy atom). Additionally, 91.1% of residues were in the most favored regions of the Ramachandran plot, and 8.4% are in the additionally allowed regions. The PDB and BMRB (Biological Magnetic Resonance Bank) codes provided for the structure deposition are 6OBK and 30,592, respectively.

### 2.2. Structural Homology

For the structural homology searches, MetaGO was used to predict Gene Ontology (GO) terms by detecting functional homologs [16]. COACH [17] and COFACTOR [18] were used to predict potential protein-ligand binding sites, Enzyme Commission numbers and GO terms. Calculation were carried locally using the standalone versions of COACH and COFACTOR distributed with I-TASSER 5.1. The final approach was to search for structural homologs directly against all PDB entries. Only entries with at least one protein of 23 to 63 residues with a minimum of three helices were considered. This initial selection resulted in 6260 PDB entries, for a total of 37,624 protein chains. Each of these structures were compared with the NMR structure of ORF47 using TM-align [19]. The optimal superposition was based on detected alignments as well as the TM-score, a metric for measuring the similarity of two protein structures. TM-scores range from 0 to 1, where a score > 0.5 indicates a similar fold.

### 2.3. Bacterial Growth Conditions

All bacterial strains, phages and plasmids used in this study are listed in Supplementary Table S1. *L. lactis* MG1363 and its derivative were grown at 30 °C in M17 broth (Oxoid) supplemented with 0.5% (*w/v*) glucose monohydrate (GM17). For solid media, 1.0% (*w/v*) agar was added to GM17 broth. *L. lactis* MG1363 were transformed by electroporation using a glycine-based protocol [20]. To maintain cloning plasmid vectors, chloramphenicol and/or erythromycin were added to a final concentration of 5 μg/mL each (Cm5 or Em5). For cloning purposes, chemically competent *E. coli* NEB5α were purchased (New England Biolabs, Whitby, ON, Canada) and transformed according to the manufacturer’s instructions. *E. coli* transformants were grown in BHI medium supplemented with 150 µg/mL erythromycin (Em150) and incubated at 37 °C with agitation. For solid media, 1.5% (*w/v*) agar was added to BHI Em150 broth.

### 2.4. Phage Propagation and Titration

Phage p2 (GenBank GQ979703) was obtained from the Félix d’Hérelle Reference Center for Bacterial Viruses (www.phage.ulaval.ca). For phage infection, growth medium was supplemented with 10 mM CaCl_2_ (GM17+Ca). Phages were amplified from frozen glycerol stocks as previously described [10]. Phage titers were usually determined by double layer plaque assays [21] with plates containing a bottom layer of solid GM17+Ca and a top layer of GM17+Ca supplemented with 0.75% (*w/v*) agar. When indicated, serial phage dilutions were spotted onto a lawn of the appropriate bacterial strain and plaques were counted at the lowest dilution at which they were visible. The phage efficiency of plaquing (EOP) was calculated by dividing the phage titer obtained on the resistant strain by the phage titer obtained on the sensitive strain. To determine if a phage resistance phenotype was due to a deficiency in adsorption, phage adsorption assays were performed as previously described [10,22]. For phage growth curves, *L. lactis* MG1363 cultures were followed for 50 min post-infection with a starting MOI of 0.05, as described elsewhere [23]. Growth curves were conducted with biological and technical triplicates. The burst size was calculated with this formula: (Average final titer—average initial titer)/average initial titer. The latent period ends with host cell lysis and was determined by measuring the time it took for the phages to reach the middle of the exponential phase. To obtain concentrated and purified phage p2∆47 for a time-course infection assay, one liter of a second amplification lysate was purified on a discontinuous CsCl gradient [24].

### 2.5. Complementation and Bacterial Gene Knockout

All primers and oligonucleotides used in this study are listed in Supplementary Table S2. To construct pNZ0677, primers were designed to clone the gene *llmg_0677* into the vector pNZ123 and were oriented so that transcription was driven from the promoter upstream of the chloramphenicol resistance gene. Primers were designed to amplify the gene of interest and add extensions overlapping pNZ123 at the XbaI restriction site for Gibson assembly [25]. The repair template pKO0676 was constructed similarly into pNZ123, with a disrupted gene *llmg_0676* (frameshift deletion of 268 bp) flanked with two homologous arms of approximately 1 kb to allow efficient recombination. Resulting plasmids were transformed into *L. lactis* MG1363 and the inserts were confirmed by sequencing using primers pNZins_F and pNZins_R.

For the knockout of *llmg_0676*, a spacer targeting this gene was cloned in the CRISPR RNA (crRNA) of pL2Cas9, transformed into the cloning host *E. coli* NEB5α, purified from overnight *E. coli* cultures using the QIAprep Spin Miniprep kit (Qiagen, Toronto, ON, Canada) and electroporated into *L. lactis* MG1363 already containing pKO0676. The presence of the correct spacer in pL2Cas9 was confirmed by sequencing PCR products using primers Cas9_F6 and crRNA_R. The gene deletion in the chromosome of *L. lactis* MG1363 was also confirmed by Sanger sequencing of PCR products.

### 2.6. DNA Sequencing and Analysis

Sanger sequencing was performed with an ABI 3730xl analyzer at the Plateforme de Séquençage et de Génotypage des génomes at the CRCHU-CHUL center. The whole genome of three BIM_p2∆47_ were sequenced using the Illumina platform. Raw sequencing reads were deposited in the NCBI SRA database under BioProject ID PRJNA645370. The genome of *L. lactis* MG1363 and one BIM_p2∆47_ were also sequenced using the PacBio platform. For whole genome sequencing, DNA extraction, library preparation, and assembly were performed as described previously [26]. For Illumina, libraries were prepared using the Nextera XT DNA library preparation kit according to the manufacturer’s instructions. Sequencing was performed on a MiSeq using a MiSeq reagent kit v2. For PacBio, the reads were assembled into a single contig according to the manufacturer’s instructions. Sequences were analyzed using Geneious 11.1.2.

### 2.7. Proteomic Analyses

The proteomic analyses of phage infections were conducted as previously described [10]. Briefly, time-course infections of *L. lactis* MG1363 by phage p2∆47 were performed in biological triplicate. Sample T0 (negative control) was collected just prior to adding the purified phage p2∆47 at a MOI of 5. Samples T10, T20 and T40 were collected 10, 20, and 40 min post-infection, respectively. Proteins were extracted and separated by 1D SDS-PAGE (12% polyacrylamide) for in-gel tryptic digestion. Tryptic peptides were separated by liquid chromatography (LC) using an EASY-nLC II system and measured on an Orbitrap Velos instrument. For GeLC-MS/MS, relative protein quantification was achieved using the MaxQuant software (version 1.6.0.1) and the Andromeda plug-in. Proteins were accepted if at least two unique peptides could be identified in at least two of the three biological replicates (filtered in Perseus, version 1.6.0.2.) (Supplementary Table S3). Statistical analysis (ANOVA) was performed using TM4 where *p*-values ≤ 0.01 were considered significant (Supplementary Table S4). For classification and visualization purposes, all *L. lactis* MG1363 proteins were assigned a TIGRFAM functional role (TIGRRole) [27].

The MS data were deposited on the ProteomeXchange Consortium via the PRIDE partner repository [28] with the dataset identifier PXD013186.

## 3. Results and Discussion

### 3.1. Sequence Homology, NMR Structure Determination, and Structure Homology

**Figure 2:**
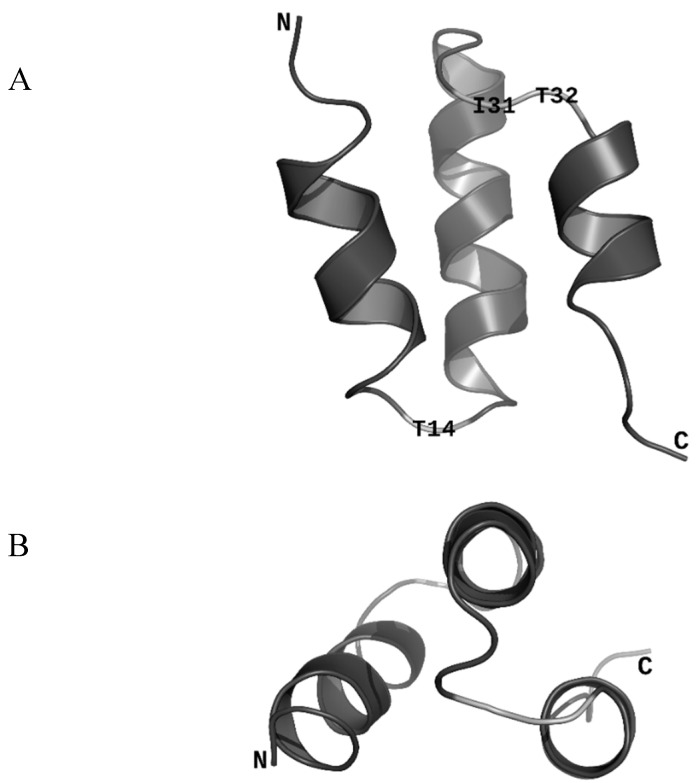
ORF47 overall fold in ribbon representation. The free amino group (N-terminus) is indicated as N. The carboxyl group (C-terminus) is indicated as C. (**A**) ORF47 representation showing the three α-helices. The three highlighted residues are the three least conserved residues among ORF47 homologues and are all located in loops. (**B**) ORF47 representation showing that two of the three α-helices are parallel. PDB ID: 6OBK.

The sequence encoding ORF47 is found in all 184 publicly available *Skunavirus* genomes (NCBI: txid1623305). All proteins are exactly 43 amino acid residues long and the sequence is extremely well conserved, which is uncommon for non-structural phage proteins (Supplementary Figure S1). This conservation suggests that ORF47 plays an important role in phage replication. Still, some variations were identified at positions 14, 31, and 32. We used NMR to determine the structure of ORF47 (PDB ID: 6OBK). The protein is generally ordered and composed of three α-helices (Figure 2). The three less conserved residues are all located in loops and are highlighted in Figure 2.

In an attempt to reveal potential functional features of ORF47, we used three different structural homology search tools, namely MetaGO [16], COACH [17], and COFACTOR [18]. These searches did not generate significant results. We then searched for structural homologs against all Protein Data Bank (PDB) entries. The most significant hit (TM-score = 0.55, Cα RMSD = 2.1 Å) was with PDB entry 2XF7 (UniProt entry O48468) [29], which corresponds to gp23.1, a protein of 51 residues belonging to SPP1, a virulent phage of the Siphoviridae family infecting the Gram-positive bacteria *Bacillus subtilis*. gp23.1 was shown to assemble in a hexamer in the crystal lattice as well as in solution and was hypothesized to act as a chaperone, possibly involved in the assembly of structural phage proteins. Despite the similar fold of gp23.1 and ORF47, it seems unlikely that the latter would have a similar function since our NMR data (peak linewidths and NOE, data not shown) and FPLC profile (data not shown) suggest that ORF47 exists as a monomer *in vitro*. Moreover, in the phage SPP1 genome, *gp23.1* is located between genes coding for tail and cell lysis proteins, whereas *orf47* is located away from the tail and lysis modules and at one extremity of the phage p2 genome.

### 3.2. Impact of orf47 Deletion on Phage Fitness

Growth curves were carried out to determine the latent period and the burst size of the mutant phage p2∆47 and the wild-type phage p2. Both phages had a 35.5-min latent period, indicating that the lack of *orf47* did not affect the duration of the lytic cycle. However, the number of infective phage particles released per infected bacterium was significantly lower with p2∆47 yielding a burst size of 80 ± 7 compared to 129 ± 17 for p2 (Supplementary Figure S4). These results show that ORF47 is not essential for phage p2 replication but is necessary for the phage optimal multiplication in *L. lactis* MG1363 under our experimental conditions.

### 3.3. Impact of orf47 Deletion on Bacterial Physiology

**Figure 3:**
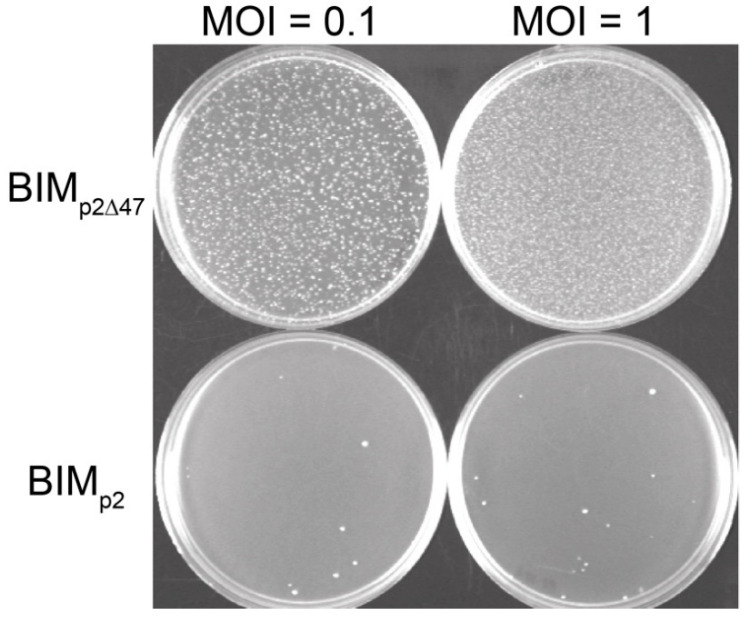
Frequency of natural BIMs generated following the infection by either phage p2 or phage p2∆47. The number of BIMs obtained following p2 infection (**bottom**) is significantly lower than the number of BIMs obtained following p2∆47 infection (**top**). This bacterial resistance to phage p2∆47 was observed at all MOIs tested (only 0.1 and 1 are shown).

We infected *L. lactis* MG1363 with phage p2 or p2∆47 and over incubated the plates to study the natural emergence of phage-resistant colonies (BIMs; bacteriophage-insensitive mutants). Our data clearly showed that fewer bacteria survived infection by phage p2 compared to infection by p2∆47 (Figure 3).

The genome of selected BIMs obtained following infection by phage p2 (BIM_p2_) were sequenced and found to be mutated at the cell surface receptor, specifically within genes responsible for the biosynthesis of a cell wall-associated polysaccharide (CWPS). CWPS-encoding genes are known to be involved in facilitating adsorption of phages belonging to the *Skunavirus* genus [30]. All analyzed BIM_p2_ were resistant to p2 and p2∆47 due to a deficiency in adsorption (data not shown).

Surprisingly, the resistance exhibited by BIMs resulting from the challenge with phage p2∆47 (BIM_p2∆47_) was not due to altered phage receptors, as revealed by phage adsorption assays and sequencing of the above-mentioned genes coding for the CWPS. These BIM_p2∆47_ showed resistance only to p2∆47 and not to the wild-type phage p2. To confirm these findings, we cloned *orf47* into an expression plasmid, introduced it in *L. lactis* MG1363 and challenged the resulting transformant with phage p2∆47. The frequency of BIMs was low and similar to the frequency of BIMs observed with the wild-type strain *L. lactis* MG1363 and the wild-type phage p2. Moreover, these BIMs were phage-receptor mutants.

Taken altogether, our findings indicate that the presence of *orf47* in phage p2 drastically reduces the natural emergence of BIMs in *L. lactis* and also affects the host response by influencing the type of resistance mechanisms.

## 4. BIM_p2∆47_ Genotype

The complete genome sequences of three BIM_p2∆47_ were obtained by Illumina sequencing. We found a 395 bp deletion perturbing gene *llmg_0677* (A2RJ28) in all three genomes while this region was not deleted in our wild-type strain. Deletions were confirmed in seven additional BIM_p2∆47_ by PCR and subsequent Sanger sequencing. To ensure that we did not miss additional mutations, the whole genomes of one BIM_p2∆47_ and the wild-type strain *L. lactis* MG1363 were obtained by PacBio sequencing. We confirmed that the only difference between BIM_p2∆47_ and our MG1363 strain was the 395 bp deletion.

**Figure 4:**
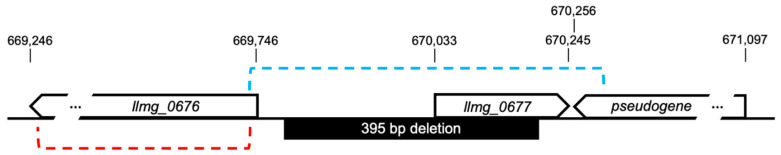
Genomic region encompassing the deletion found in BIM_p2∆47_. The dashed blue line indicates the region cloned into pNZ123 for complementation. The dashed red line indicates the region deleted for gene inactivation.

The 70-amino acid protein encoded by *llmg_0677* is predicted to have two alpha-helical transmembrane regions and is likely embedded in the cell membrane but has no putative functions. We attempted to restore the sensitivity of the BIM_p2∆47_ to p2∆47 by complementing the deletion (including the whole *llmg_0677*) (Figure 4) using pNZ123 as an expression vector. While we were able to clone the deleted fragment, the assay failed to restore the phage sensitivity phenotype, suggesting that genomic context for the expression of this probable membrane protein is important.

Next, we hypothesized that the 395 bp deletion might interfere with the expression of gene *llmg_0676* encoding a hypothetical acetyltransferase (A2RJ27) located 56 bp upstream of the deletion (Figure 4). Using CRISPR-Cas9, we knocked out gene *llmg_0676* and the expected deletion in the resulting clone MG∆0676 was confirmed by PCR and sequencing. We then checked for phage resistance by comparing the p2 and p2∆47 titers on the recombinant strain MG∆0676 and on the sensitive *L. lactis* MG1363. The phage titers were the same with both bacterial strains, implying that the inactivation of *llmg_0676* is not responsible for the resistance to phage p2∆47 in BIM_p2∆47_. The mechanisms by which BIM_p2∆47_ develop resistance to phage infection remain to be elucidated.

### Impact of ORF47 on Bacterial Proteomes

To determine the impact of ORF47 on bacterial cells, we analyzed the proteomic signature resulting from the infection of *L. lactis* MG1363 by phage p2∆47 and compared it with previous data obtained with the wild-type phage p2 [10]. Of note, both datasets rely on the same control (same *L. lactis* MG1363 uninfected cultures) but separate searches were performed for calculation of label-free quantification (LFQ) intensities. LFQ intensities are normalized on multiple levels to take into account one whole dataset and ensure that profiles of LFQ intensities across samples accurately reflect relative protein abundances. Because the normalization might be slightly different between the two datasets (p2 and p2∆47), we do not directly compare LFQ intensities. Instead, to compare p2 and p2∆47 infections, we rely on the fold changes (infection vs. control) calculated for each of the datasets, separately.

**Figure 5:**
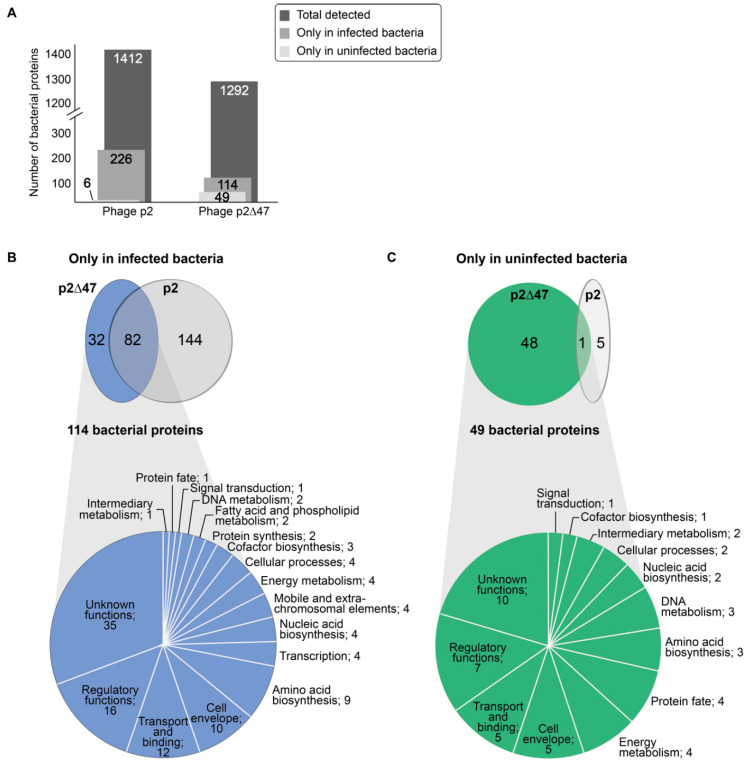
*L. lactis* MG1363 proteins identified during time-course infections by phage p2 and phage p2∆47. (**A**) Bar graph comparing the number of bacterial proteins detected during the infection of *L. lactis* MG1363 by either phage p2 or phage p2∆47. (**B**) Venn diagram depicting the overlaps of bacterial proteins identified strictly in infected cultures (not at T0) or (**C**) identified strictly in uninfected cultures (only at T0). Pie charts illustrate the proportion and the classification of these bacterial proteins according to their role(s) in metabolic pathways for the dataset generated with phage p2∆47. In (**B**) and (**C**), the number of proteins is indicated after the classification.

Interestingly, deletion of the gene coding for the low-abundance protein ORF47 had a major impact on the bacterial proteome during phage infection. Previously, 1412 bacterial proteins were detected in our proteomic analysis of *L. lactis* MG1363 [10]. Among these proteins, 226 were detected only during phage p2 infection while 6 were detected strictly in uninfected cultures [10]. Here, a total of 1292 bacterial proteins were detected with phage p2∆47 infecting *L. lactis* MG1363. The number of bacterial proteins detected only in p2∆47-infected *L. lactis* MG1363 cultures fell to 114, while the number of bacterial proteins detected only in uninfected cultures rose to 49 (Figure 5A).

Among the 114 bacterial proteins detected strictly during p2∆47 infection, 82 were shared by both phage p2∆47 and phage p2 infection (Supplementary Table S5). The remaining 32 bacterial proteins were unique to phage p2∆47 infection (Supplementary Table S6). Interestingly, 144 *L. lactis* MG1363 proteins were detected only during infection by phage p2, but not by phage p2∆47 (Supplementary Table S7) (Figure 5B). Collectively, these data suggest that ORF47 inhibits as well as triggers the synthesis of many bacterial proteins during phage infection.

Of the 49 proteins detected solely in the uninfected cultures in the *L. lactis* MG1363 and phage p2∆47 proteomic analysis, only one protein, A2RJ92 (DexC protein, neopullulanase), is also present strictly in the uninfected cultures from the phage p2 analysis [10]. The 48 other proteins were detected in both the uninfected and infected cultures of the p2 dataset (Figure 5C and Supplementary Table S8).

**Figure 6:**
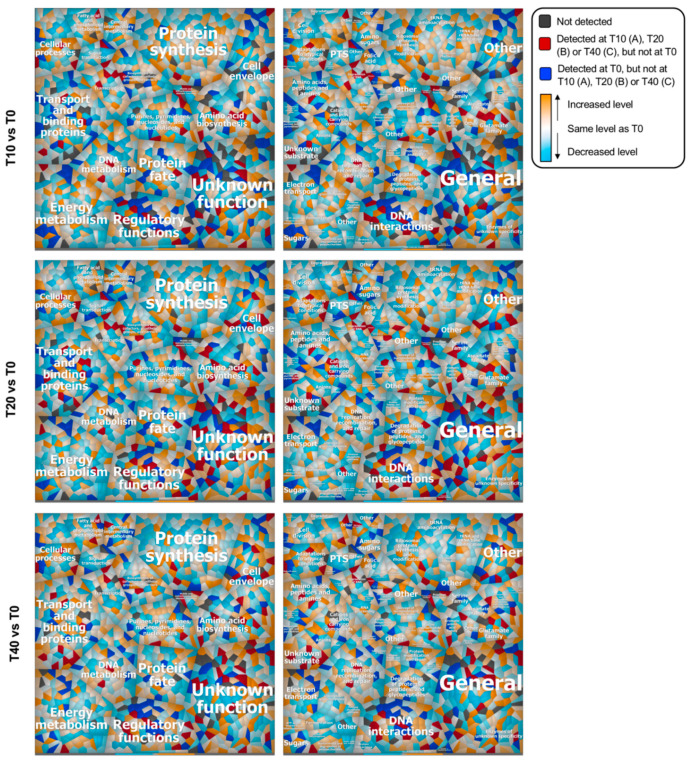
Voronoi treemaps depicting the proteotypes of *L. lactis* cells during phage p2∆47 infection. Each cell represents a single gene locus grouped with other functionally related elements. Metabolic pathways assigned to gene loci are based on TIGRRoles (left panel) and TIGR subroles (right panel). Proteins were determined at 10 (T10), 20 (T20) and 40 (T40) minutes post-infection and compared with uninfected cultures (T0). Expression data were visualized using a color gradient. Colors of the range blue means a protein expression level lower than in uninfected cultures (T0), white means equal to T0, and orange means higher than T0. For higher resolution images of the subroles treemaps (right panel), see Figures S5–S7 (Supplementary Materials).

To compare the *L. lactis* MG1363 proteomic response to phage p2 or p2∆47 infection, we built Voronoi treemaps and compared the fold changes (control versus infection) calculated for each of the datasets. These treemaps allow the linking of functional classification to the relative bacterial protein changes during phage infection. They also facilitate the comparison and comprehensive representation of processes taking place in infected cells. We expected to see an increase in most functional categories as was previously observed throughout the infection by phage p2 (10). Instead, a global decrease was observed in all major metabolic pathways during the infection with phage p2∆47, illustrated by a predominance of blue Voronoi cells (Figure 6).

The bacterium dormancy-like state might explain the reduced burst size of phage p2∆47 compared with phage p2. Still, we observed increases for proteins involved in cellular processes (subrole adaptations to atypical conditions) and amino acid biosynthesis (subrole glutamate family) at all time points after infection, illustrated by the number of orange and red Voronoi cells in these subcategories. The concentration of proteins associated with transport and binding (subrole unknown substrate) was also higher 40 min post-infection.

**Figure 7:**
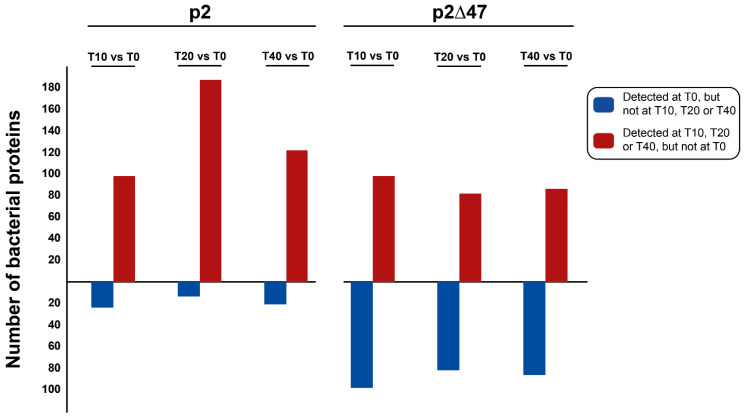
Bar graph depicting the number of bacterial proteins detected only in uninfected cultures (dark blue) or only during infection (dark red). The number of proteins is indicated on the *y*-axis. There are significantly more genes that are induced than genes that are inhibited by phage p2 infection (**left**). In comparison, there are fewer genes induced, and more genes inhibited following phage p2∆47 infection (**right**).

In Figure 6, proteins that were only detected at specific stages of infection (T10, T20 or T40) are represented by dark red cells and the proteins that were only detected in the uninfected cultures (T0) are represented by dark blue cells. The abundance of these proteins in the p2∆47 dataset differ sharply from the p2 dataset (Figure 7) and are scattered in all functional categories (Figure 6), suggesting that ORF47 induces and suppresses the expression of many genes that are not functionally related.

## 5. Conclusions

At the beginning of infection, phages hijack their host’s cellular machinery to create a favorable environment for their own propagation. For the vast majority of phage–host pairs, the bacterial pathways that are altered and the phage proteins that are responsible for these metabolic shifts are unknown. In this study, we characterized ORF47, a low-abundance small phage protein. With Voronoi treemaps, we provided an overview of the processes in the cell at the protein level during phage p2 infection in the absence of its ORF47. By comparison with our previous work [10] we found that this phage protein activates the cellular machinery to prevent a dormancy-like state in the bacterial host during phage infection. Our results suggest that ORF47 controls a wide variety of processes in the host and as such, may act as a regulator. Most notably, we showed that the viral protein ORF47 affects the bacterial response by blocking the rapid emergence of phage resistance, as shown by the significantly higher frequency of BIMs generated following the infection by phage p2∆47. The underlying mechanisms by which ORF47 hinders phage resistance remain unclear. From a biotechnological standpoint, because phage therapy can result in the emergence of phage-resistant bacteria, the discovery of phage proteins blocking BIM generation could be valuable for such application.

## Supplementary Materials

Supplementary materials can be found at https://www.mdpi.com/1999-4915/12/8/797/s1.
