# Supplementary material for: A Lactococcal Phage Protein Promotes Viral Propagation and Alters the Host Proteomic Response During Infection
Source: Viruses. 2020 Jul 24;12(8):797. doi: 10.3390/v12080797 (PMC7472136; doi:10.3390/v12080797)
Supplement: Supplementary file 1 [file viruses-12-00797-s001.zip › SuppMat.pdf]

SUPPLEMENTARY FIGURES, TABLES AND REFERENCES

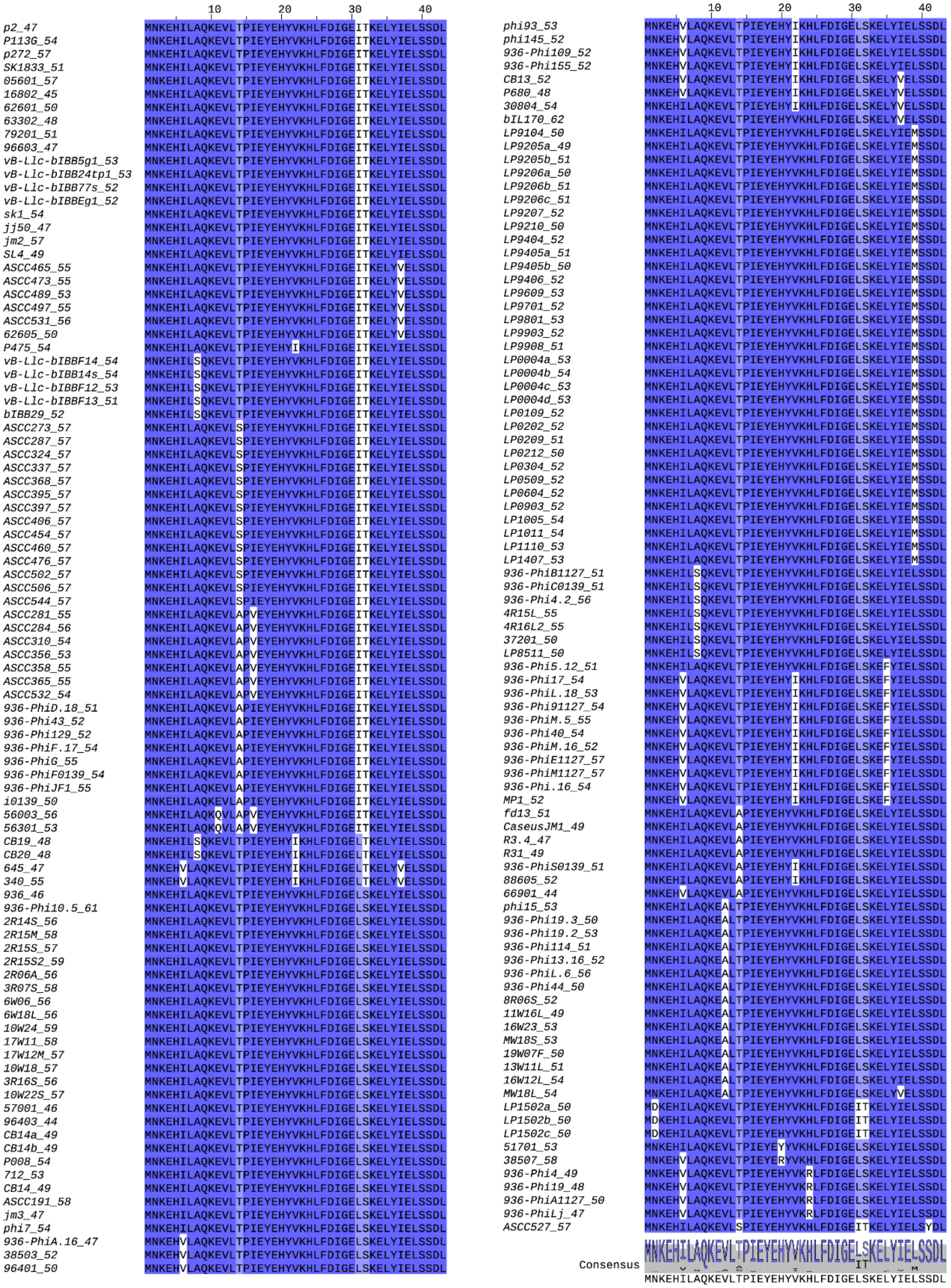

Figure S1. Sequence alignment of ORF47 with equivalent proteins in 184 phages of the *Skunavirus* genus. ORF47 (p2_47) is the first protein of the list. The consensus sequence is showed at end of the list.

Figure S2. 2D homonuclear NMR spectra of ORF47. (A) 2D COSY in D_2O. (B) "Fingerprint region" of 2D COSY in H_2O. (C) 2D TOCSY. (D) 2D NOESY with 200 ms mixing time.

See PNG file *SupplementaryFigureS2*

Figure S3. Natural abundance 2D heteronuclear NMR spectra of ORF47. (A) ^{13}C-HSQC (full spectra, with folding of CH_3 region). (B) ^{13}C-HSQC (^{1}Hα-^{13}Cα expansion). (C) ^{15}N-HSQC.

See PNG file *SupplementaryFigureS3*

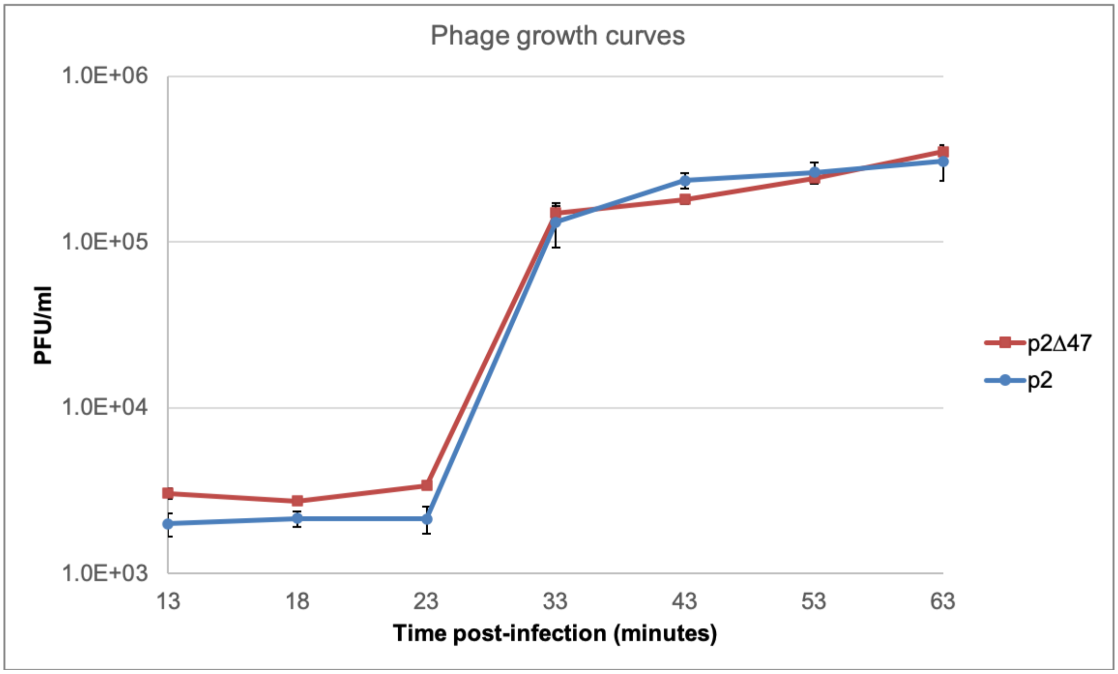

Figure S4. Growth curves of phage p2 (blue) and p2Δ47 (red) using *L. lactis* MG1363 as a host. The dots represent the average of technical and biological triplicates. Error bars are shown.

Figure S5. Voronoi treemap depicting the proteotype of *L. lactis* cells 10 minutes post-infection by phage p2Δ47. Metabolic pathways assigned to gene locus are based on TIGR subroles.

See PNG file *SupplementaryFigureS5*.

Figure S6. Voronoi treemap depicting the proteotype of *L. lactis* cells 20 minutes post-infection by phage p2Δ47. Metabolic pathways assigned to gene locus are based on TIGR subroles.

See PNG file *SupplementaryFigureS6*.

Figure S7. Voronoi treemap depicting the proteotype of *L. lactis* cells 40 minutes post-infection by phage p2Δ47. Metabolic pathways assigned to gene locus are based on TIGR subroles.

See PNG file *SupplementaryFigureS7*.

Table S1. Bacterial strains, phages and plasmids used in this study.

See Excel file *SupplementaryTables*.

Table S2. Primers and oligonucleotides used in this study.

See Excel file *SupplementaryTables*.

Table S3. *L. lactis* MG1363 and phage p2Δ47 protein identifications.

See Excel file *SupplementaryTables*.

Table S4. Log-2 transformed label-free quantification (LFQ) intensities for *L. lactis* MG1363 proteins detected in this study. The averaged values of LFQ intensities were calculated only if at least two unique peptides could be identified in at least two of the three biological replicates.

See excel file *SupplementaryTables*.

Table S5. *L. lactis* MG1363 proteins detected strictly during infection by both phage p2 and phage p2Δ47. Proteins are classified according to their TIGR roles and subroles.

See Excel file *SupplementaryTables*.

Tables S6. *L. lactis* MG1363 proteins detected strictly during infection by phage p2Δ47. Proteins are classified according to their TIGR roles and subroles.

See Excel file *SupplementaryTables*.

Table S7. *L. lactis* MG1363 proteins detected strictly during infection by phage p2. Proteins are classified according to their TIGR roles and subroles.

See Excel file *SupplementaryTables*.

Table S8. Bacterial proteins detected strictly in uninfected cultures in the proteomic analysis of *L. lactis* MG1363 and phage p2Δ47, and detected during the infection by phage p2. Proteins are classified according to their TIGR roles and subroles.

See Excel file *SupplementaryTables*.

Supplementary References

31. Gasson MJ. 1983. Plasmid complements of *Streptococcus lactis* NCDO 712 and other lactic streptococci after protoplast-induced curing. J Bacteriol 154: 1–9.
32. Higgins DL, Sanozky-Dawes RB, and Klaenhammer TR. 1988. Restriction and modification activities from *Streptococcus lactis* ME2 are encoded by a self-transmissible plasmid, pTN20, that forms cointegrates during mobilization of lactose-fermenting ability. J Bacteriol 170: 3435–3442.
33. De Vos WM. 1987. Gene cloning and expression in lactic streptococci. FEMS Microbiol Lett 46: 281–295.
