# Supplementary figures and images for: A Lactococcal Phage Protein Promotes Viral Propagation and Alters the Host Proteomic Response During Infection
Source: Viruses. 2020 Jul 24;12(8):797. doi: 10.3390/v12080797 (PMC7472136; doi:10.3390/v12080797)

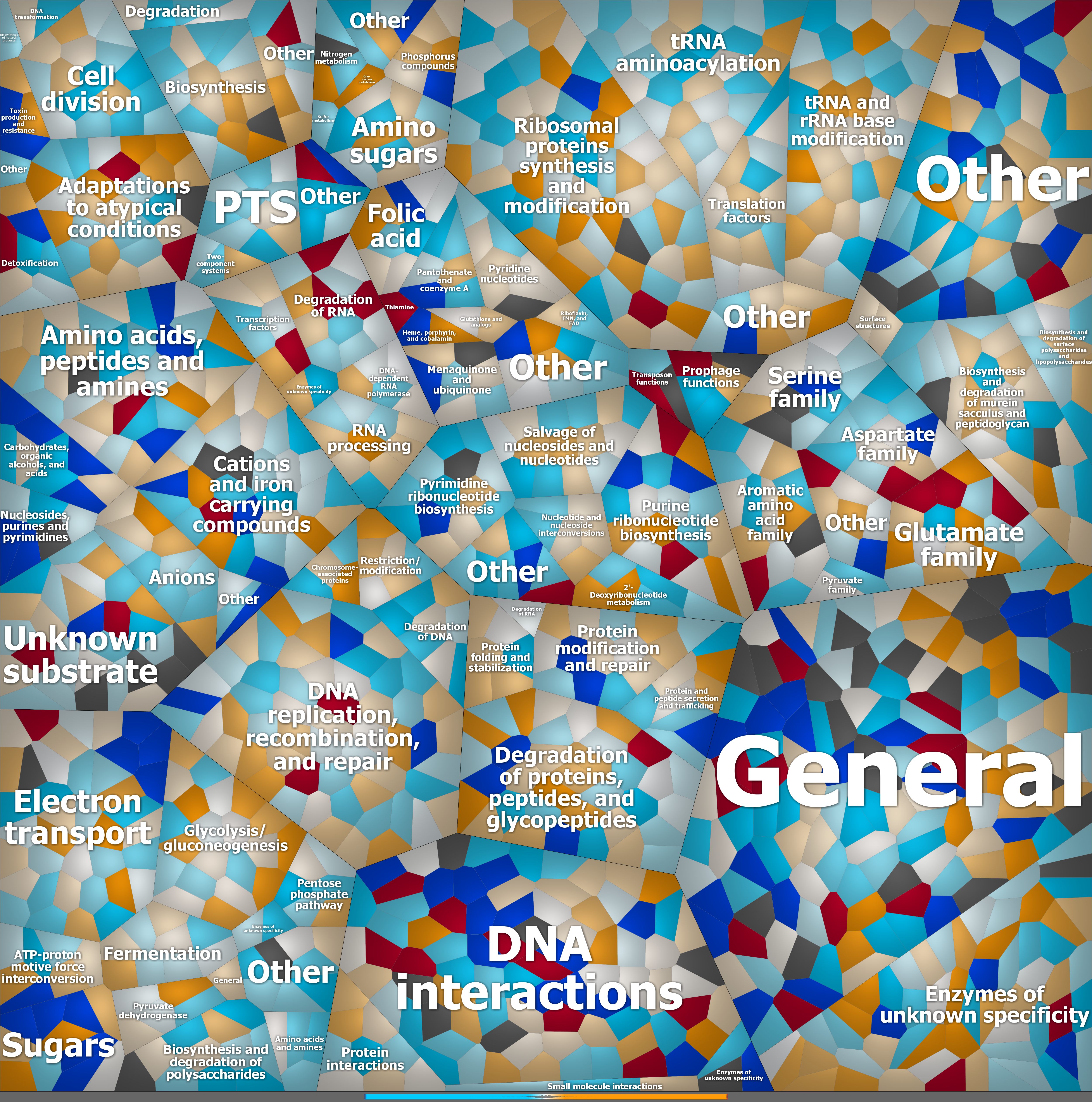

Supplement: Supplementary file 1 [file viruses-12-00797-s001.zip › SupplementaryFigureS5.png]

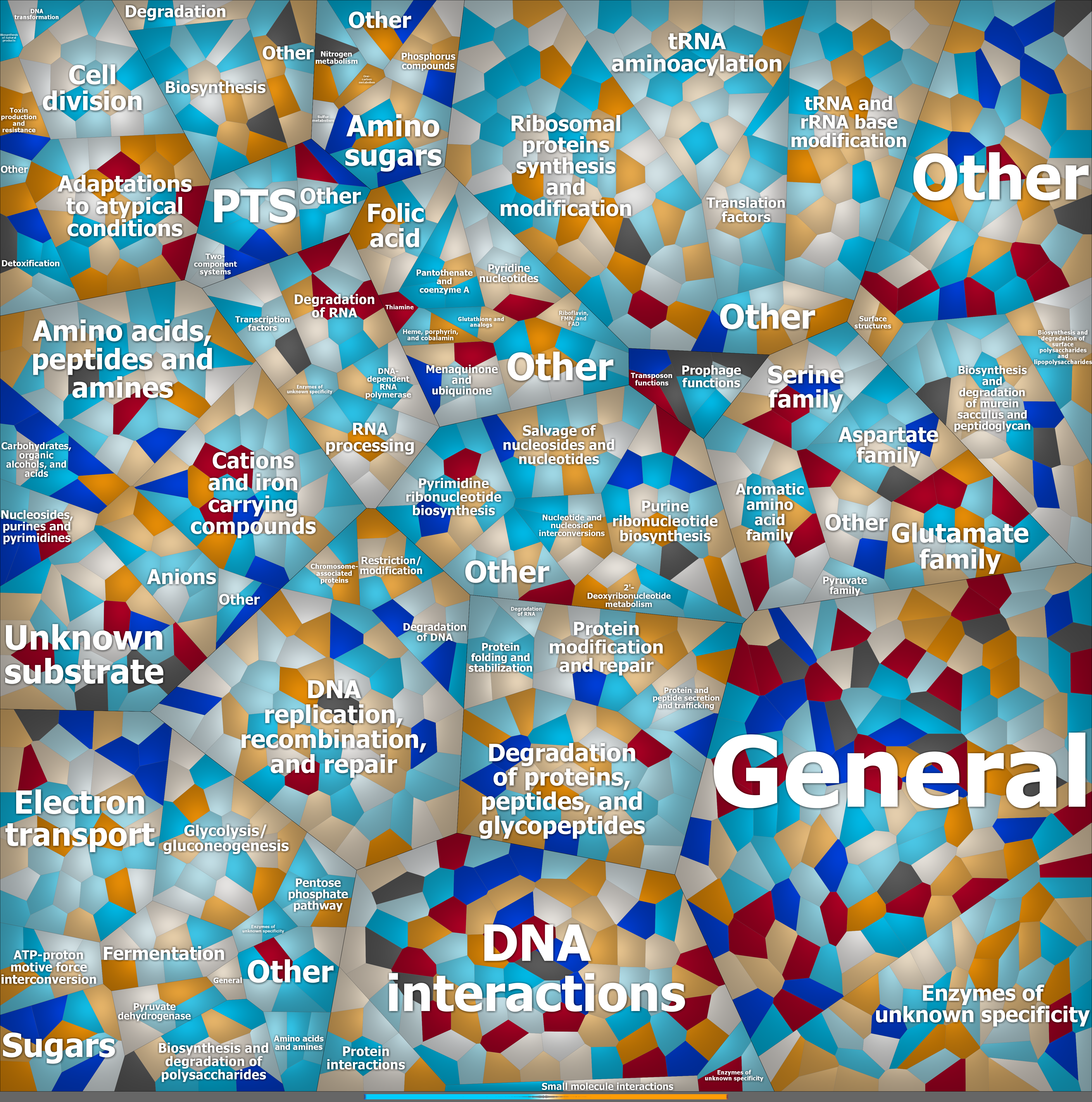

Supplement: Supplementary file 1 [file viruses-12-00797-s001.zip › SupplementaryFigureS6.png]

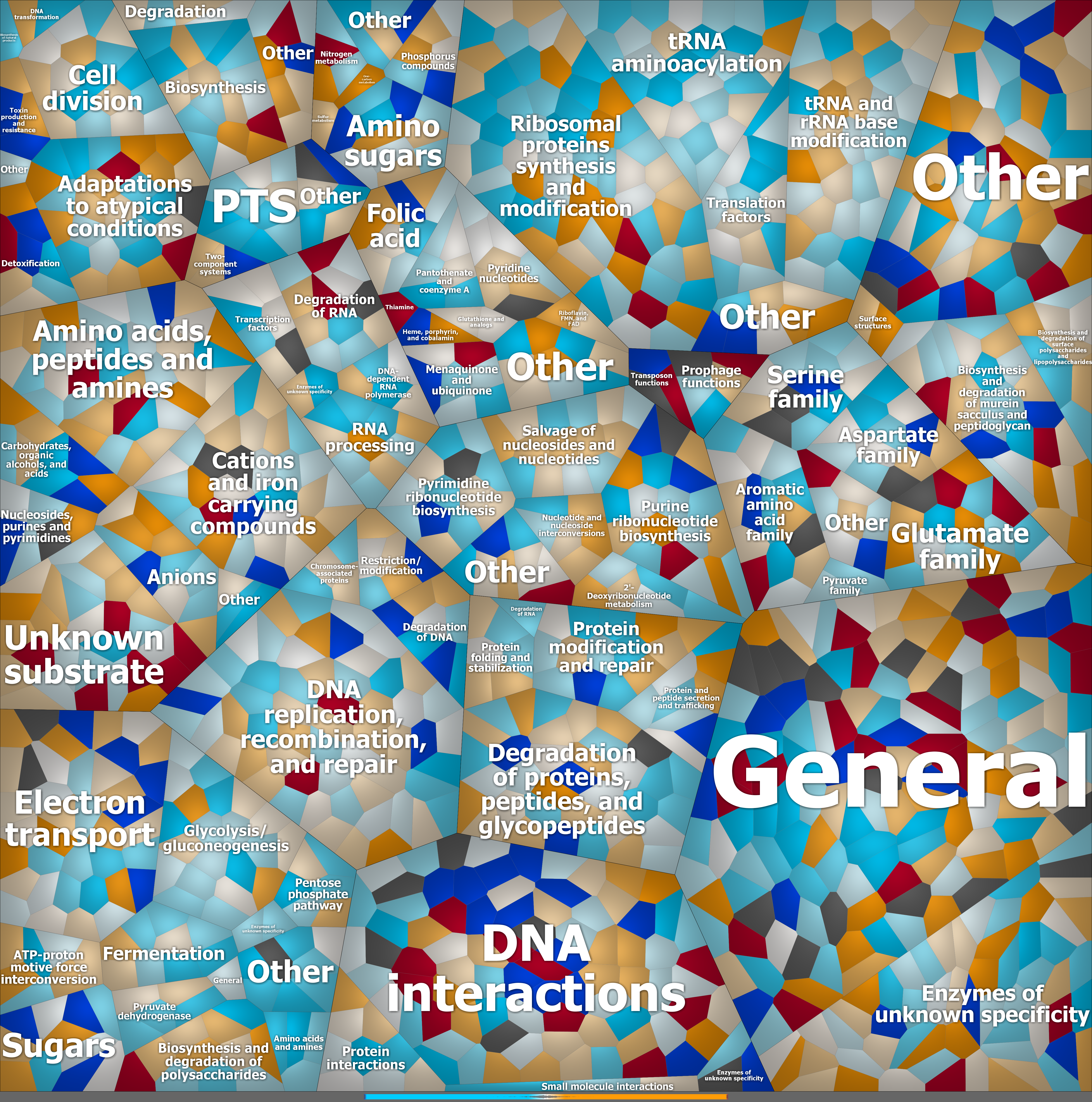

Supplement: Supplementary file 1 [file viruses-12-00797-s001.zip › SupplementaryFigureS7.png]

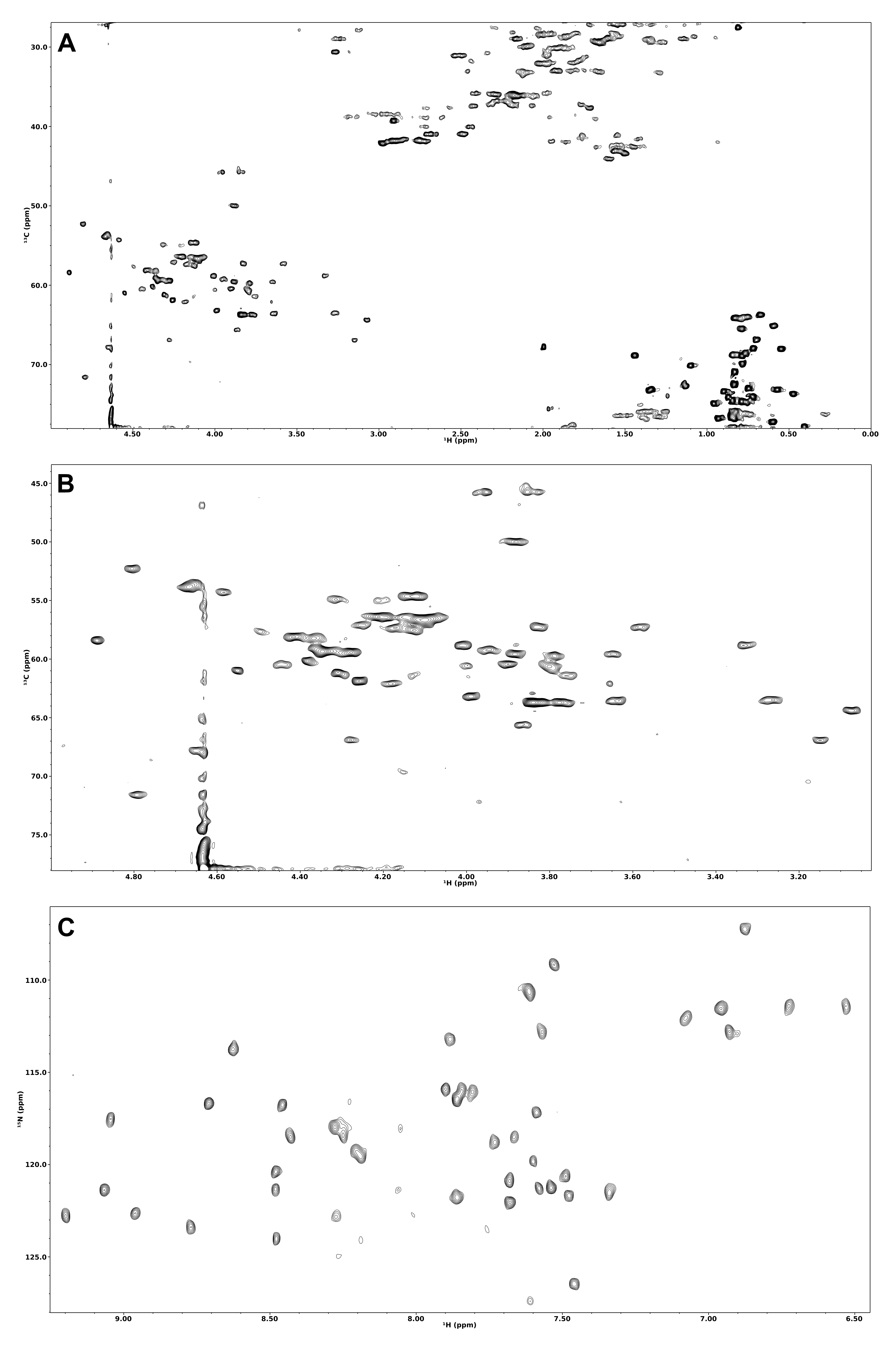

Supplement: Supplementary file 1 [file viruses-12-00797-s001.zip › SupplementaryFigureS3.png]
